# Peripheral immune circadian variation, synchronisation and possible dysrhythmia in established type 1 diabetes

**DOI:** 10.1007/s00125-021-05468-6

**Published:** 2021-05-18

**Authors:** Craig A. Beam, Eleni Beli, Clive H. Wasserfall, Stephanie E. Woerner, Megan T. Legge, Carmella Evans-Molina, Kieran M. McGrail, Ryan Silk, Maria B. Grant, Mark A. Atkinson, Linda A. DiMeglio

**Affiliations:** 1 Department of Biomedical Sciences, Homer Stryker MD School of Medicine, Western Michigan University, Kalamazoo, MI USA; 2 Present Address: Wellcome Wolfson Institute for Experimental Medicine, Queens University Belfast, Belfast, NI UK; 3 Indiana University Center for Diabetes and Metabolic Diseases, Indianapolis, IN USA; 4 Department of Pediatrics, Indiana University School of Medicine, Indianapolis, IN USA; 5 Department of Pathology, Immunology and Laboratory Medicine, University of Florida, Gainesville, FL USA; 6 University of Florida Diabetes Institute, Gainesville, FL USA; 7 Richard L. Roudebush VA Medical Center, Indianapolis, IN USA; 8 Department of Ophthalmology, University of Alabama, Birmingham, AL USA

**Keywords:** Circadian rhythms, Clinical, Immune cells, Type 1 diabetes

## Abstract

**Aims/hypothesis:**

The circadian clock influences both diabetes and immunity. Our goal in this study was to characterise more thoroughly the circadian patterns of immune cell populations and cytokines that are particularly relevant to the immune pathology of type 1 diabetes and thus fill in a current gap in our understanding of this disease.

**Methods:**

Ten individuals with established type 1 diabetes (mean disease duration 11 years, age 18–40 years, six female) participated in a circadian sampling protocol, each providing six blood samples over a 24 h period.

**Results:**

Daily ranges of population frequencies were sometimes large and possibly clinically significant. Several immune populations, such as dendritic cells, CD4 and CD8 T cells and their effector memory subpopulations, CD4 regulatory T cells, B cells and cytokine IL-6, exhibited statistically significant circadian rhythmicity. In a comparison with historical healthy control individuals, but using shipped samples, we observed that participants with type 1 diabetes had statistically significant phase shifts occurring in the time of peak occurrence of B cells (+4.8 h), CD4 and CD8 T cells (~ +5 h) and their naive and effector memory subsets (~ +3.3 to +4.5 h), and regulatory T cells (+4.1 h). An independent streptozotocin murine experiment confirmed the phase shifting of CD8 T cells and suggests that circadian dysrhythmia in type 1 diabetes might be an effect and not a cause of the disease.

**Conclusions/interpretation:**

Future efforts investigating this newly described aspect of type 1 diabetes in human participants are warranted. Peripheral immune populations should be measured near the same time of day in order to reduce circadian-related variation.

**Graphical abstract:**

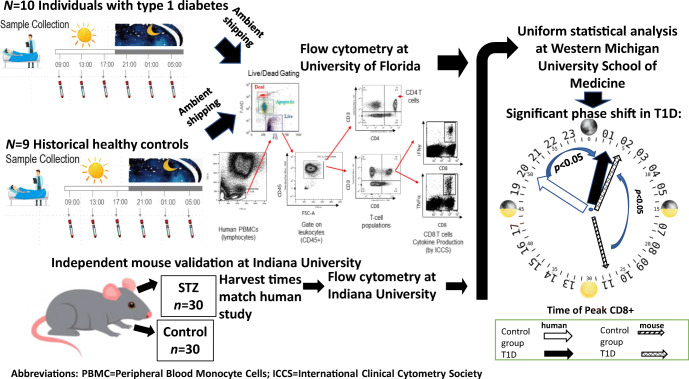

**Supplementary Information:**

The online version contains peer-reviewed but unedited supplementary material available at 10.1007/s00125-021-05468-6.

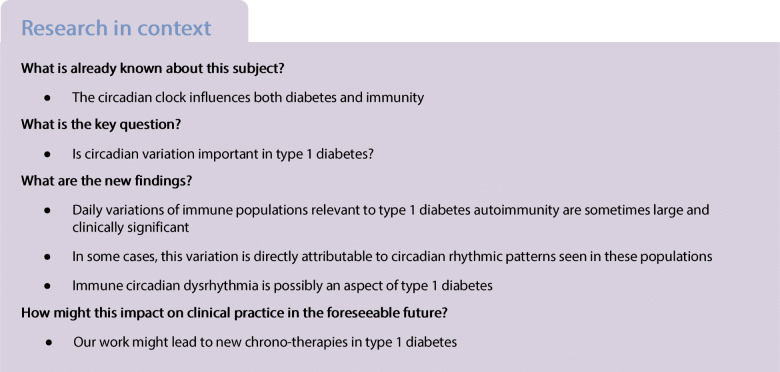



## Introduction

The circadian clock, a system of oscillating neuroendocrine hormonal signalling and cellular transcription factors distributed throughout the body, coordinates human physiology with the daily light/dark cycle, optimising organismal physiologic processes [1]. The clock is an important aspect of diabetes as well: it is essential for proper metabolic control and regulation of beta cell function [2] and the pancreatic islet transcriptome exhibits circadian variation [3]. Both insulin secretion and insulin action exhibit circadian patterns [4]. Qian et al [5] found that mice exposed to constant light had diminished glucose-stimulated insulin secretion due to a decrease in insulin secretory pulse mass. Marcheva and colleagues [6] found that conditional ablation of the pancreatic circadian clock caused diabetes in mice due to defective beta cell function during the late stage of stimulus–secretion coupling. Recently it was shown that, in human type 2 diabetes, alpha and beta cells have attenuated Clock expression disrupting insulin and glucagon releases [7].

Indeed, circadian disruption or dysrhythmia is associated in humans with diabetes and its complications as well [8]. Leproult et al [9] found that the circadian misalignment occurring in shift workers increases diabetes risk through mechanisms not related simply to sleep loss. These mechanisms likely involve the dysregulation of hormones and energetics as the circadian clock machinery controls and is itself controlled by both hormones and metabolic status [10–12]. Using murine diabetic models, circadian dysregulation has been associated with the appearance of complications such as peripheral neuropathies [11], accelerated progression of diabetic complications [13, 14] and reduced efficacy of immunotherapies [15].

The circadian clock is also an important and scientifically well-established aspect of immunity [16, 17]. Circadian patterns of immunity in humans have been previously described [18, 19] and peripheral circadian clocks have been found to regulate immune cell trafficking from peripheral blood to tissues, including the secondary lymph system [20]. Circadian-induced variation of some of the immune populations thought to be involved in type 1 diabetes has been previously described elsewhere [19, 21–27]. However, these studies were conducted in healthy people, were not specific to type 1 diabetes and were characterisations of broad populations, e.g. CD4 and CD8 T cells, and not of population subsets and cytokines that are components of type 1 diabetes autoimmunity (e.g. central memory, effector memory and regulatory T cell [Treg cell] subpopulations). We sought to address this gap in knowledge and more thoroughly characterise the circadian variation and rhythmicity in peripheral blood cell populations thought relevant to, or reflective of, the autoimmune process involved in type 1 diabetes and to compare these patterns with those previously observed in healthy control (HC) groups. We also sought to expand this circadian characterisation to include cytokines in order to capture a greater understanding than has heretofore been accomplished. Finally, we sought to fill another shortcoming in the literature by seeking to analyse and interpret our mechanistic findings in a clinically meaningful context.

## Methods

### Study participants

Participants were enrolled at the Indiana University (IU) School of Medicine. After informed consent, participants underwent a medical history check and physical examination. Participants were between 18 and 40 years old, had type 1 diabetes for at least 12 months without any other clinically significant diseases or illnesses, weighed at least 110 pounds (49.9 kg) and were willing to provide written informed consent, abstain from alcohol, maintain a regular sleep schedule for 1 week prior to the day of the trial and spend 24 h in the research facility in compliance with study requirements. Participants were excluded if they did shift work that required changes in sleep patterns, if they had travelled between time zones within 1 week prior to the study or if they were taking any immune modulatory medications. The IU Institutional Review Board approved all study procedures.

### Study protocol

**Fig. 1:**
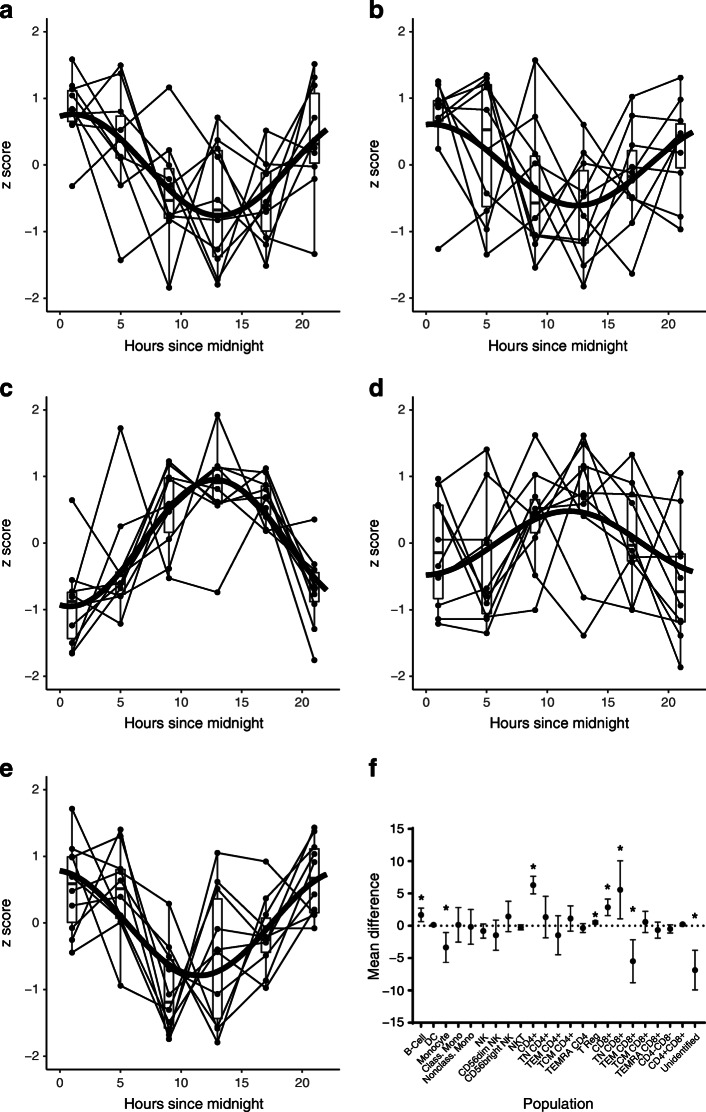
Circadian patterns in type 1 diabetes. (**a**–**e**) Daily variations observed in immune populations that have statistically significant circadian rhythmicity. A statistically fitted circadian curve is superimposed on data from each participant. Box-plots summarise data at each sampling time point, *N*=10. (**a**) B cells, (**b**) DCs, (**c**) effector memory CD4, (**d**) effector memory CD8 and (**e**) Treg . (**f**) Mean within-subject difference (95% CI) in cell populations sampled at 01:00 minus 09:00 hours in type 1 diabetes patients. Statistically significant differences (*p* < 0.05) are indicated by asterisks. Class. mono., classical monocytes; Nonclass. mono., non-classical monocytes; TN, naive T cells; TEM, effector memory T cells; TCM, central memory T cells

Participants were admitted to the Indiana Research Center between 07:00 and 08:00 hours on the day of the study. Participants were allowed an unrestricted diet during the study, including caffeinated beverages. Lights were turned off at 23:00. Blood samples were obtained via venipuncture at (±30 min) 09:00, 13:00, 17:00 and 21:00 hours on day 1, and 01:00 and 05:00 hours on the following day. Blood samples were aliquoted, with one sample processed immediately at each collection time at the IU Research Center (‘fresh’) and the other shipped (ambient) to University of Florida (UF) (‘shipped’) for processing. The shipped samples were analysed at UF in order to compare the participants with type 1 diabetes against HC samples that had been collected earlier and which had also been shipped (ambient) to UF (HC data reported by Beam et al [28]). Both the type 1 diabetes and HC samples were processed under the same protocol using the same cytometer and by the same UF personnel. All samples were handled in accordance with IU guidelines and regulations. Participants were discharged after the final sample collection. A schematic of the study design is shown in electronic supplementary material (ESM) Fig. 1.

### Flow cytometry

All whole blood (200 μl) samples (HC, fresh type 1 diabetes and shipped type 1 diabetes) were stained with the same antibody cocktail designed to provide a broad profile of immune cell subsets [29].

All antibodies were purchased from Biolegend (San Diego, CA, USA) unless indicated otherwise: APC/H7-CD3 (SK7, BD Biosciences, San Jose, CA, USA), BB515-CD45RA (HI100, BD Biosciences), PE-Cy7-HLA-DR (LN243), BV650-CD4 (RPA-T4), BV711-CD8 (SK1), BV510-CD14 (M5E2), PerCPCCy5.5-CD16 (3G8), AlexaFluor647-CD25 (BC96), BV605-CD56 (HCD56), PE-CD127 (A019D5) and BV-421 CD197 (G043H7). Antibodies were mixed in appropriate concentrations in BD Horizon Brilliant stain buffer (BD Biosciences) and 50 μl was added for each sample and fluorescence minus one (FMO) control samples. Samples were incubated for 30 min at room temperature and protected from light. Then, 2 ml of Fix/Lyse 1X (eBioscience, San Diego, CA, USA) was added and incubated at room temperature for 5 min. Two successive washing/centrifugation (5 min, 450 *g*) steps were performed with BD FBS stain buffer (BD Biosciences). The pellet was resuspended in 300 μl of BD FBS stain buffer and stored at 4°C. Samples were acquired within 24 h on a BD Fortessa cytometer with the same configurations at the Flow Cytometry Resource Facility at IU and data were analysed by FlowJo (FlowJo, Ashland, OR, USA) using the same template as for the HC samples that were also processed at UF.

### Cytokines

Plasma was isolated from EDTA tubes by centrifugation with the remaining sample after flow cytometry aliquots were dispensed. This was then immediately frozen until batch analyses using Luminex (Austin, TX, USA) technology and kits from Millipore (Burlington, MA, USA) as previously described [28].

### Clock genes

Total RNA was isolated from each Tempus Blood RNA tube using the Tempus Spin RNA isolation kit (ThermoFisher, Waltham, MA, USA). RNA was reverse transcribed using the High-Capacity RNA-to-cDNA kit (ThermoFisher). Relative gene expression was quantified using a custom TaqMan array using validated gene expression assays (ESM Table 1).

### Murine validation experiment

Male C57BL6 mice were obtained from the Jackson Laboratory (Bar Harbor, ME, USA) at 8 weeks of age. Mice were housed in the institutional animal care facilities at the IU Medical School with a strict 12 h:12 h light/dark cycle. Diabetes was induced with an injection of streptozotocin (STZ) (50 mg/kg). Animals were confirmed to be diabetic after 1 month of housing and when the serum glucose level was above 13.9 mmol/l for at least two consecutive measurements. At 4 months of age, diabetic and control mice were randomly assigned to a time point of a 24 h cycle and terminated at ZT (‘zeitgeber’; hours since ‘lights on) times ZT1, ZT5, ZT9, ZT13, ZT17 and ZT21. Single-cell suspensions were prepared from whole blood at the termination of the experiment. Samples were fixed and data were acquired on an LSR II flow cytometer (Beckman Coulter Life Sciences, Indianapolis, IN, USA). Further details are provided in the ESM Methods.

### Statistical methods

We followed study design protocols and analysis methodologies that are commonly found in human circadian studies, including sample size selection. In addition, we adjusted for individual participant-level differences in the dynamic range of immune factors via standardisation prior to analysis and adjusted for individual variation in the timing of individual participant-level circadian rhythms by incorporating the simultaneous measurement of cortisol in our statistical models. Statistical methods for systems immunology analysis of the data follow those used previously by the team [28]. Further details can be found in the ESM Methods.

## Results

### Demographics

Ten individuals with type 1 diabetes were recruited for the study. Six (60%) were female and four (40%) were male. Mean age was 27 years (range: 18–40 years). Average time post diagnosis was 11 years (minimum 1 year). A comparison with the baseline characteristics of the HC individuals is provided in ESM Table 2.

### Several immune populations and serum cytokine levels exhibit appreciable daily variation and biologically significant circadian rhythmicity in type 1 diabetes patients with established disease

Daily variation (the daily difference between highest and lowest levels or ‘daily range’ when comparing the six timepoint measurements for an individual) in adaptive immune populations was often found to be appreciable (Table 1). B cells (expressed as percentage lymphocytes) varied by as much as 13.8 percentage points within one individual. For the average individual, B cells varied by 4%. In one individual, the CD4 effector memory subpopulation varied by 10.4%, while for another individual CD8 effector memory varied by as much as 31.9%. The average high–low differences for these populations were 5.8% and 10.7%, respectively. In contrast, CD4 Treg cells exhibited much less daily variation. The maximum range observed within a single participant was a daily difference of 2.4% with an average of 1.0%. Similarly, natural killer (NK) cells of the innate immune system were also found to be highly variable, with one participant having a daily difference of 9.4% (average difference=4.7%). On the other hand, dendritic cells (DCs) were found to have minor variation: the maximum daily difference was 0.7% with an average of 0.34%. The majority of the immune cells measured in circulation also exhibited a statistically significant circadian cosine pattern (Table 2).

**Table 1:** Daily ranges of immune population frequencies and plasma cytokine levels in ten participants with type 1 diabetes

Population	Minimum	Maximum	Mean	ICC
Leucocytes (%)				
Granulocyte	8.4	43.3	19.36	43%
Monocytic leucocytes	8.3	42.2	19.3	43%
Lymphocytes (%)				
B cell	1.2	13.8	4.02	50%
DC	0.2	0.7	0.34	53%
Monocyte	2.9	11.2	7.93	69%
Classical monocyte	3.9	12.8	7.82	49%
Non-classical monocyte	3.9	12.8	7.82	49%
NK	1.2	9.4	4.65	21%
CD56^dim^	2.6	11.8	6.49	71%
CD56^bright^	2.6	11.8	6.49	71%
NKT	0.2	5.0	0.99	69%
CD4 T cell	5.3	26.6	11.41	19%
Naive	2.5	11.7	7.38	94%
Effector memory	0.9	10.4	5.79	92%
Central memory	1.5	9.7	5.56	97%
TEMRA	0.1	5.3	0.93	59%
Treg	0.4	2.4	0.95	52%
CD8 T cell	1.7	15.3	5.64	72%
Naive	3.3	27.1	12.07	85%
Effector memory	3.6	31.9	10.69	71%
Central memory	1.2	6.6	3.47	95%
TEMRA	1.0	6.9	2.78	96%
CD4^−^CD8^−^	0.5	4.0	1.39	81%
CD4CD8	0.1	0.7	0.34	61%
Unidentified	7.3	66.9	20.42	0%
Plasma cytokines (pg/ml)				
IFNγ	0.4	1.7	1.08	83%
IL-2	0.0	1.5	0.62	84%
IL-4	2.0	11.9	6.12	89%
IL-6	1.1	5.2	2.21	73%
IL-12	0.1	0.9	0.37	80%

For each component, data presented are minimum, maximum and mean of the ranges found across all time points in each of the participants

ICC, intraclass correlation

**Table 2:** Adaptive and innate immune population circadian peak level and time of peak in participants with type 1 diabetes

Immune cell population	Circadian rhythmicity	Peak (SD units)	95% CI	Time of peak^a^	95% CI
Granulocytes	†‡	0.67	0.4015, 0.9699	12:54	10:48, 14:24
Monocytic/lymphocytes	†‡	0.68	0.4245, 0.9868	00:24	00:04, 23:54
B cells	†‡	0.76	0.5116, 1.0415	01:06	00:09, 23:48
DCs	†‡	0.61	0.3484, 0.9135	00:42	00:08, 23:54
Monocytes	†‡	0.51	0.2350, 0.8465	10:24	07:48, 12:56
Classical monocytes	†‡	0.75	0.5147, 1.0212	03:54	02:24, 5:30
Non-classical monocytes	†‡	0.75	0.5073, 1.0218	15:54	14:24, 17:30
NK cells		0.34	0.1028, 0.6932	13:30	08:42, 17:36
CD56^dim^ NK cells		0.37	0.1126, 0.7228	15:05	10:48, 18:57
CD56^bright^ NK cells		0.36	0.1082, 0.7175	03:06	00:24, 23:04
NKT cells	†	0.45	0.1899, 0.7773	15:18	12:12, 16:12
CD4 T cells	†‡	0.89	0.6418, 1.1502	00:18	00:02, 23:57
CD4 naive T cells	†‡	0.74	0.4818, 1.0243	02:36	01:12, 4:12
CD4 effector memory T cells	†‡	0.48	0.2039, 0.8316	11:48	08:58, 14:30
CD4 TEMRA		0.12	0.0453, 0.5306	11:36	22:24, 1:24
CD4 central memory T cells		0.23	0.0618, 0.6129	16:24	02:54, 22:48
Treg cells	†‡	0.79	0.5150, 1.0817	23:30	00:03, 23:58
CD8 T cells	†‡	0.86	0.6293, 1.1150	01:04	00:06, 23:36
CD8 naive T cells	†‡	0.93	0.6974, 1.1822	01:06	00:12, 2:12
CD8 effector memory T cells	†‡	0.95	0.7250, 1.1971	12:48	11:54, 13:36
CD8 TEMRA		0.30	0.0891, 0.6603	14:54	08:54, 19:03
CD8 central memory T cells		0.31	0.0979, 0.6590	16:54	12:06, 21:24
CD4^−^CD8^−^ T cells		0.16	0.0495, 0.5478	15:18	02:06, 22:30
CD4CD8 T cells	†‡	0.50	0.2205, 0.8425	00:03	23:54, 00:05
Unidentified cells	†‡	0.71	0.4408, 1.0231	13:30	12:06, 15:00

^a^24 h clock time

^†^Statistically significant, *p* < 0.05

^‡^Statistically significant at 10% FDR; see ESM Table 4

Inspection of circadian patterns (Table 2 and Fig. 1) confirms a well-known ‘chrono-phenotype’ demarcating two major subgroups of immune cell populations: one group that peaked during the ‘night’ (18:00 to 06:00 hours; Fig. 1a,b,e) and one group that peaked during the ‘day’ (06:00 to 18:00 hours; Fig. 1c,d). Night-peaking cell populations were lymphocytes, B cells, DCs, naive CD4 and CD8 T cells, Treg cells and classical monocytes. Day-peaking cell populations were granulocytes, non-classical monocytes, natural killer T (NKT) cells, effector memory CD4 and CD8 T cells, and other unidentified cells.

Among the plasma cytokines, IL-4 exhibited the largest daily difference (Table 1): one individual experienced a high–low difference of 11.99 pg/ml. The average daily difference of 6.12 pg/ml for this cytokine exceeded that of all other cytokines. Contrasted with IL-12, having an average daily difference of 0.37 pg/ml, the difference in variability with IL-4 is appreciable. Although only Il-6 was found to have statistically significant circadian rhythmicity, all cytokines were estimated to peak during the early evening (i.e. 17:00 to 21:00 hours) (Table 3).

**Table 3:** Plasma cytokine circadian peak level and time of peak in participants with type 1 diabetes

Plasma cytokine	Circadian rhythmicity	Peak (SD units)	95% CI	Time of peak^a^	95% CI
IFNγ		0.20	0.01, 0.42	17:45	04:39, 23:38
IL-2		0.21	0.01, 0.43	17:14	04:45, 23:06
IL-4		0.15	0.01, 0.30	18:14	02:46, 23:50
IL-6	†	0.51	0.23, 0.85	20:22	17:30, 22:30
IL-12		0.33	0.15, 0.78	18:07	09:20, 22:29

^a^24 h clock time

^†^Statistically significant, *p* < 0.05

In summary, daily ranges of immune variables in type 1 diabetes patients were large and several immune populations and cytokines thought to be relevant to type 1 diabetes exhibited statistically significant circadian rhythmicity.

### Biologically and clinically significant circadian peak–trough differences

On the basis of our circadian pattern analysis we observed that, in general, peaks and troughs occur near 09:00 and 01:00 hours. We then analysed the within-subject differences (01:00 vs 09:00 hours) in each of the cell populations measured in this study (Fig. 1f). We first observed that in several populations these approximate peak and trough values differ significantly statistically from zero (*p*<0.05), suggesting that circadian variation is not inconsequential fluctuation, but is indicative of true biologically significant changes (i.e. true change in mean population frequency) in cell population frequencies during the day.

Second, we observed that some of the differences are of a magnitude that is considered clinically significant as well. For example, Herold and colleagues [30] found that a reduction as small as 1.7% in baseline CD8 effector memory populations discriminated participants treated with teplizumab (anti-CD3mAb) who subsequently ‘responded’ (preserved beta cell function) from those who did not, although they were also given the active treatment. As can be observed from Fig. 1f, CD8 effector memory and several other immune populations had peak–trough differences of this magnitude or larger. Thus, circadian variation can also be of a clinically significant magnitude.

### Peripheral immune synchronisation

Correlations among the immune factors are presented in ESM Fig. 2. The data are ordered by acrophase (or time of peak) starting at 6:00 hours, which was approximately the time of daylight on the day of the study. Importantly, this arrangement of the data shows the succession in peak occurrence so that immune factors that peak after another factor appear sequentially and, along with the identification of biologically significant and false discovery rate (FDR)-significant correlations, documents the extent of phase synchronisation in the peripheral immune system. We define a ‘biologically significant’ correlation to occur whenever variation in one immune factor accounts for more than 10% of the variation seen in another factor, as indicated by the square of the correlation between them (known as ‘*r*^2^’ or ‘coefficient of determination’). An FDR-significant correlation is one that is statistically significant after controlling the FDR to be no greater than 10% among the many correlations generated by our data (see ESM Methods).

The 28 sequentially ordered immune factors give rise to 27 peak-adjacent correlations of which eight (30%) meet the criteria for biological and FDR significance, thereby establishing phase synchronisation between the following pairs: NK, CD8 T-effector memory cells (r=0.55); CD8 T-effector memory CD45RA-expressing cells (TEMRA), NK CD56^dim^ (r=0.73); NKT, CD4^−^CD8^−^ (r=0.69); CD4 central memory T cells, CD8 central memory T cells (r=0.54); IL-4, IFNγ (r=0.62); IL-4, IL-6 (r=0.57); B cells, CD8 (r=0.52); classical monocytes, NK56^bright^ (r=0.54).

**Fig. 2:**
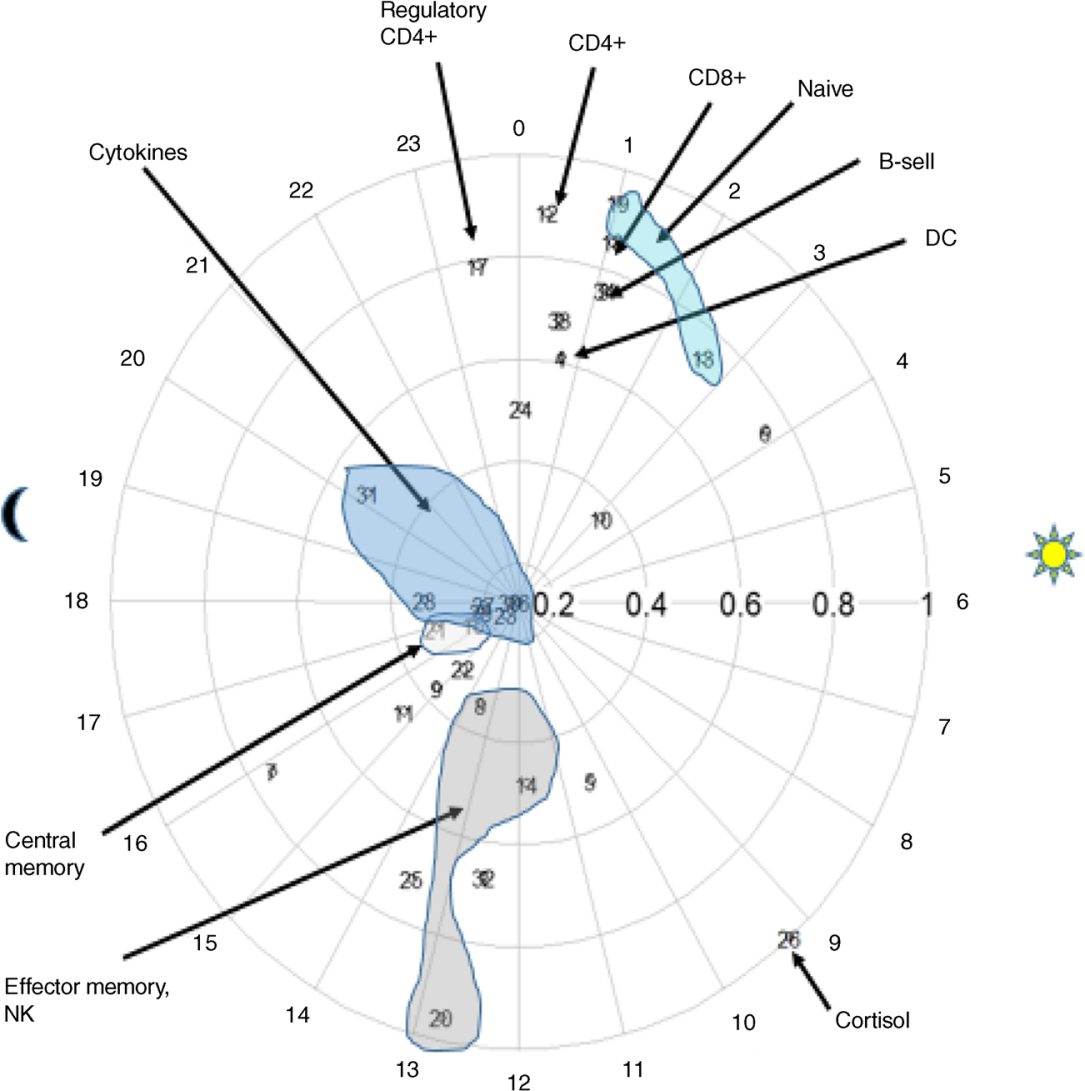
Functional synchronisation. Chronograph of immune components measured in type 1 diabetes (shipped samples). Each numbered circle represents an immune component. Placement of the circle on the 24 h ‘clock’ indicates acrophase (time of peak). Amplitude, or height of peak (expressed in standard deviation units), is indicated by distance from the clock centre. Times of sunrise and sunset on the day of the study are indicated by sun and moon symbols. Some data points have been visually grouped by functional phenotype

Further insight into synchronisation within the peripheral immune system in type 1 diabetes is given by Fig. 2, in which time of peak (‘acrophase’) and peak level (‘amplitude’) of the fitted circadian patterns are plotted on a 24 h clock face. From this figure it can be observed that phase synchronisation occurs within functional phenotypes.

### Circadian rhythmicity of the Clock gene transcriptome

Clock genes are found in most cells and express proteins which act as transcription factors, producing circadian rhythmicity in cellular functioning via negative feedback looping. For example, clock gene *PER* (for Period) is a repressor of the expression of the transcription activator CLOCK/BMAL1 heterodimer [1]. Circadian variation in expression of the transcription factor PER, due to entrainment of the organism to external variation in light, is then thought to drive circadian oscillation in transcription activators CLOCK (circadian locomotor output cycles protein kaput) and BMAL1 [1] (brain and muscle ARNT [aryl hydrocarbon receptor nuclear translocator]-like protein 1). ESM Table 3 reports analysis of circadian rhythmicity in clock gene transcripts measured from peripheral whole blood sampled from the participants with type 1 diabetes. Seven (64%) of the 11 clock genes measured in this study displayed significant circadian rhythmicity.

### Comparison with HC individuals

Data from a historical group of ten healthy individuals (six male participants, age: 22–38 years) who previously had participated in a circadian sampling study [28] were used for comparison. The HC samples had been shipped to the UF for processing and FACS analysis. These data were compared with aliquoted samples from participants with type 1 diabetes which had also been shipped to UF and processed in the same way as the HC samples (see Methods).

Table 4 reports the results of comparing the circadian features of the type 1 diabetes and historical HC cohorts (note that no cytokine comparisons were made because only one cytokine was found to have significant circadian rhythmicity, which was no longer significant after adjusting for false discovery). Differences in acrophase and their associated 95% CIs suggest that statistically significant (*p* < 0.05) phase shifts (CI does not include the value zero) occurred in the time of peak occurrence of B cells (+4.8 h from that observed in HC samples), CD4 and CD8 T cells (~+5 h) and their naive and effector memory subjects (~+3.3 to +4.5 h), and Treg cells (+4.1 h).

**Table 4:** Circadian amplitude and acrophase differences between immune populations of HC samples and type 1 diabetes patients (shipped samples)

Population	Amplitude			Acrophase		
	T1D–HC	95% CI		T1D–HC	95% CI	
Granulocytes	−0.26	−0.73	0.2	3.06†	0.34	5.36
Monocytic/lymphocytes	−0.24	−0.71	0.23	3.18†	0.48	5.39
B cells	0.04	−0.43	0.5	4.76†	2.46	7.62
DCs	0.16	−0.24	0.63	3.78	−3.18	12.14
Monocytes	−0.2	−0.71	0.31	−0.9	−5.42	3.16
Classical monocytes	0.24	−0.17	0.68	5.31†	1.34	8.14
Non-classical monocytes	0.24	−0.18	0.66	5.32†	1.46	8.12
NK cells	−0.07	−0.54	0.38	3.96	−4.44	9.22
CD56^dim^ NK cells	0.12	−0.4	0.56	4.14	−9.69	10.08
CD56^bright^ NK cells	0.12	−0.39	0.57	4.15	−9.82	10.14
NKT cells	0.11	−0.39	0.65	4.21	−1.32	7.35
CD4 T cells	0.25	−0.17	0.69	5.38†	2.75	7.76
CD4 naive T cells	−0.14	−0.51	0.18	4.54†	2.44	6.56
CD4 effector memory T cells	−0.33	−0.77	0.05	3.32†	0.42	6.03
CD4 TEMRA	−0.06	−0.53	0.39	1.08	−4.35	8.59
CD4 central memory T cells	−0.08	−0.52	0.25	−8.45	−12.0	3.23
Treg cells	0.32	−0.15	0.8	4.1†	1.10	6.98
CD8 T cells	0.06	−0.33	0.44	5.28†	3.42	7.11
CD8 naive T cells	0.23	−0.19	0.63	3.77†	1.75	5.4
CD8 effector memory T cells	0.33	−0.08	0.74	4.27†	1.59	6.71
CD8 TEMRA	0.01	−0.43	0.44	5.6	−7.18	11.7
CD8 central memory T cells	−0.18	−0.67	0.25	3.31	−8.63	9.96
CD4^−^CD8^−^ T cells	−0.3	−0.77	0.17	6.76	−7.67	11.69
CD4CD8 T cells	0.14	−0.37	0.54	4.73†	0.11	11.28
Unidentified cells	0.18	−0.27	0.64	8.12†	5.21	10.96

^†^Statistically significant difference, *p* < 0.05, according to 95% CI

T1D–HC, difference between samples from patients with type 1 diabetes and HC samples

### Validation in the mouse STZ diabetes model

**Fig. 3:**
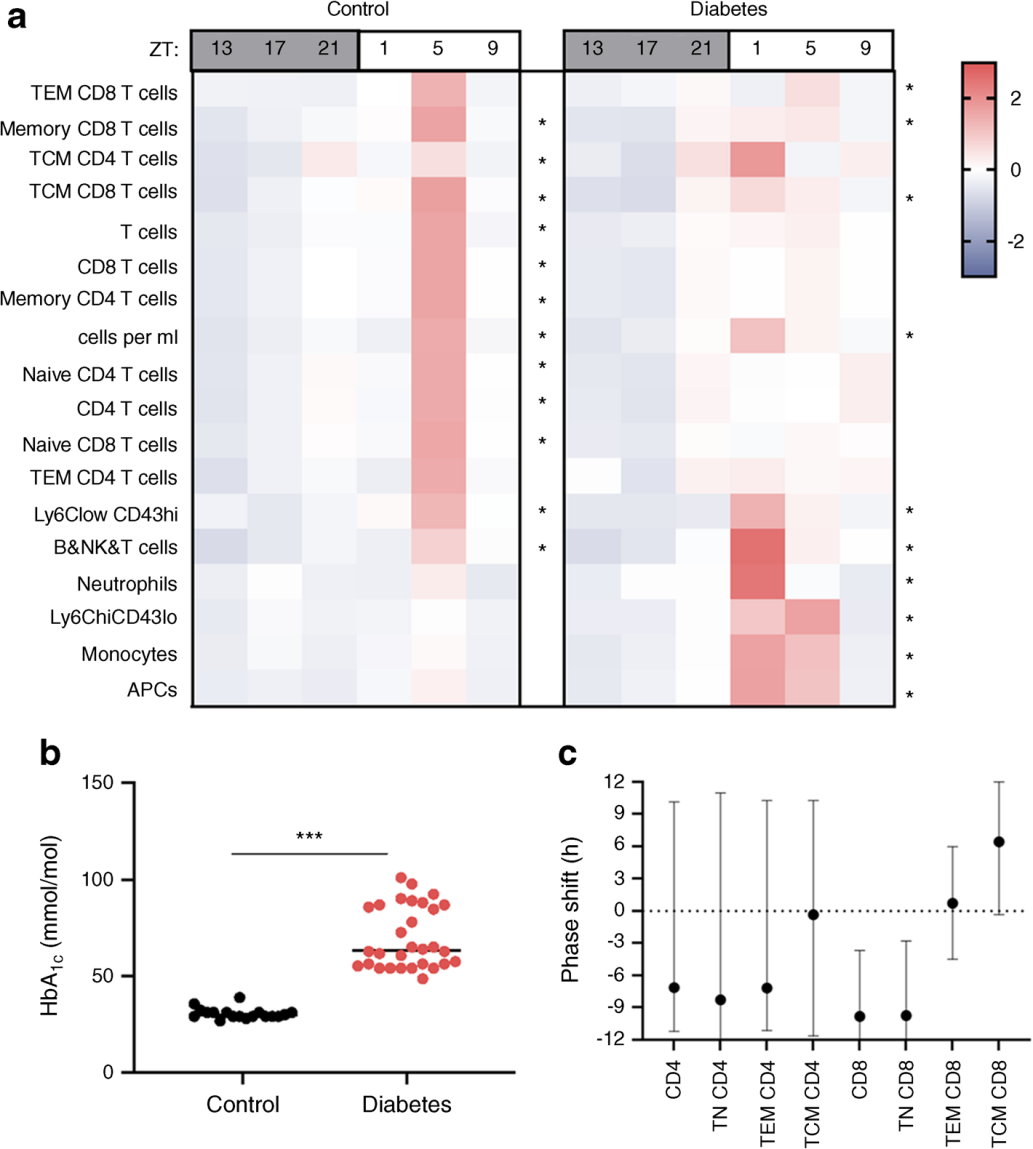
Dysrhythmia in the murine type 1 diabetes model. (**a**) Heatmap of *z* scores of cell population counts in blood from control (left) and diabetic (right) mice comparing circadian variation between the two. Asterisks indicate significant circadian rhythmicity, *p < 0.05. (**b**) HbA_1c_ levels in STZ-induced diabetes. (**c**) Phase shift in STZ-induced diabetes compared with control mice. Data are mean phase difference (diabetic−control) and 95% interval on the difference using percentage data. TEM, effector memory T cells; TCM, central memory T cells; APC, antigen-presenting cells

Results from an independent validation experiment using the STZ mouse model (see Methods) are presented in Fig. 3. Heatmaps of mean count levels across time (Fig. 3a) provide evidence of statistically significant circadian rhythmicity in both control and diabetic mice. As observed in our human study, statistically significant phase shifting was observed in the CD8 T cell population in diabetic mice (Fig. 3c). The extent of diabetes in the STZ cohort is depicted in Fig. 3b, which confirms that this experiment represents circadian aspects of autoimmune diabetes in the mouse model.

## Discussion

Circadian disruption has been found to be associated with diabetes and its complications [14, 31] as well as other autoimmune diseases such as arthritis [32] and lupus [33]. Our study now provides preliminary evidence of circadian dysrhythmia in type 1 diabetes. Importantly, significant dysrhythmia was observed in the CD8, CD4 and CD4 Treg cell populations for which, in all cases, the peak occurred significantly later in the day than was observed in HC samples.

In our parallel murine experiment, a significant phase shift was also observed in CD8 T cells. Since the STZ murine model reflects diabetes arising from induced pancreatic damage and subsequent altered immunity and glucose metabolism, we hypothesise that type 1 diabetes immune dysrhythmia is likely a result of, and not an early initiator of, this autoimmune disease.

Circadian ‘phase shifting’ has been implicated in other diseases [34] and so our discovery is supported by the literature. Yet, given the limitations of our study, further research is needed to challenge our finding of dysrhythmia before it is investigated as a possible actor in type 1 diabetes autoimmunity.

Although our study is not the first to characterise circadian rhythmicity in the human immune system, to the best of our knowledge, it is the first to provide such characterisation in immune populations and cytokine levels in a type 1 diabetes population. Moreover, our focused analysis of daily variations led to our novel discovery that circadian variation is not only biologically evident, but also is of a magnitude that has been determined to be clinically significant in some scientific investigations of therapies designed to intercede in type 1 diabetes disease progression. Importantly, we determined that mean daily peak–trough differences in CD8 effector memory populations are at a magnitude similar to the baseline difference between clinical ‘responders’ and ‘nonresponders’ to teplizumab as reported by Herold et al [30]. This finding raises a very important question related to baseline stratification efforts aimed at selecting individuals who are most likely to respond for either clinical trials or, eventually, for patient-tailored immunotherapies: if individuals with type 1 diabetes experience a daily change in CD8 effector memory populations as great as that determined to be associated with positive clinical response, how could single measurements taken at arbitrary points in time be maximally informative? Additionally, we hypothesise that the accumulated or continuous level of ‘exposure’ to immune populations across the day could improve our immune profiling at baseline and permit improved prediction of treatment response. Also, as we start to recognise and characterise endotypes of type 1 diabetes, differences in circadian patterns may identify an important new phenotype.

Druzd and colleagues [20] observed that adaptive immune responses to immunisation and pathogens depend on the time of day of exposure to the immunogen. Previous human studies also suggest that, due to circadian rhythmicity of immune populations, the timing of therapies can modulate the effectiveness of immune therapies [35]. For example, Long et al [36] show that administration of hepatitis A and flu vaccines, respectively, in the morning yielded the highest antibody titres compared with at other times. In a study of the immunosuppressive mycophenolate mofetil (MMF) in Wistar rats, Dridi et al [37] found that the level of renal toxicity was a function of the time of day of administration. This latter finding is of particular importance to type 1 diabetes since MMF has been previously tested in combination with daclizumab as a treatment for recent-onset type 1 diabetes [38]. Some therapies may be most effective when they are given in a way that maximises their effect on cell populations that are circulating; others may be better given when immune cells are resident in lymphatic tissues. Since our study has established the existence of circadian rhythmicity in immune factors considered relevant to the autoimmune process in type 1 diabetes, we therefore urge that further research be conducted of the efficacy of chrono-therapeutic approaches to immune modulation therapies.

In addition, since our data show that circadian variation leads to biologically and clinically significant differences in biomarker levels during the day, we observe that selection of the timing of biomarker measurement could modulate the range of possible immune responses. In addition, peripheral immune populations should be measured near the same time of day in order to reduce circadian-related variation and improve accuracy in the determination of responses to therapy.

Our finding of circadian rhythmicity in the clock gene transcriptome is in keeping with previous literature and, hence, provides another degree of external validation to our study. Previous murine models established the circadian rhythmicity of *PER* [39] and other clock genes [40], and our study has confirmed these findings in components of human whole blood.

Importantly, we also provide confirmatory evidence of significant circadian rhythmicity in human circulating cells of the clock genes *CLOCK* and *ARNTL*, whose mutations have been linked to the development of diabetes in mice [6]. Moreover, Lebailly and colleagues [41, 42] hypothesised that the clock gene *ARNTL2* specifically is a candidate gene for type 1 diabetes as it controls expression of the *IL21* gene, and the IL-21 cytokine is necessary for the development of diabetes in the NOD mouse [43]. We believe our work supports further investigation into this hypothesis.

Besides the use of historical control samples and the absence of technical replication to control variability, our study was limited in its precision to estimate the time of peak occurrence. Many of the CIs are wide, with some nearly covering a 12 h period. Since we based our sample size determination on previous studies, we conclude that more than ten participants are needed in order to ensure that adequate statistical precision is achieved in circadian studies of human immunity, even though ten participants proved sufficient to detect circadian rhythmicity in many populations and cytokines. Another limitation comes from our enrolment of individuals with established disease and who, therefore, have had long exposure to exogenous insulin and altered metabolism, each of which might alter circadian rhythmicity. Further research is needed to determine if there were confounding factors in our findings and interpretations. However, after adjusting for false discovery, we did not find sex or cortisol level to significantly alter circadian patterns. In addition, we note that the times of peak cortisol level were almost identical between the HC and type 1 diabetes cohorts. It is also very important to recognise that our findings are based on measurements taken from the peripheral immune system and that we have not directly measured the populations that are residing in lymph nodes or their rhythmic recruitment to pancreatic islets. Yet, recent research suggests that the day- and night-peaking immune compartments could be reciprocating manifestations of circadian-timed lymph trafficking [20], and so our observations might be translatable to the lymph system by ‘reflection’. Finally, we note that we used shipped samples in our comparison of HC and type 1 diabetes patterns, and differences in shelf-time or other factors related to shipping between the two cohorts might have confounded our results. A comparison using fresh samples might yield different results. For example, the mouse study used fresh samples and supported our human sample observation of phase shifting in CD8 populations. Yet, the mouse peak shifted in the opposite direction (earlier than control). Although this might be related to differences in the antibody panels themselves, we nonetheless recommend use of fresh samples from contemporary control groups to follow up on our initial finding in humans. However, future research into other methods of whole blood preservation could be conducted to find a substitute for fresh samples that also preserves the circadian architecture.

In conclusion, daily variation of immune populations relevant to type 1 diabetes autoimmunity is significant biologically and, in some cases, clinically. Often these variations are circadian in nature. Peripheral immune populations should be measured near the same time of day in order to reduce circadian-related variation. There is a possibility that type 1 diabetes is associated with circadian dysrhythmia which itself might be the result of metabolic (e.g. early morning dysglycaemia [44]) and immunologic perturbations related to the disease. Future efforts investigating this newly described aspect of type 1 diabetes are warranted.

## Supplementary Information

ESM 1 (PDF 641 kb)

## Data Availability

Data are available by request to the authors.

## Abbreviations

DC: Dendritic cell
FDR: False discovery rate
HC: Healthy control
IU: Indiana University
MMF: Mycophenolate mofetil
NK: Natural killer (cell)
NKT: Natural killer T (cell)
STZ: Streptozotocin
Treg: Regulatory T (cell)
TEMRA: T-effector memory CD45RA-expressing (cell)
UF: University of Florida
ZT: ‘Zeitgeber’: hours since ‘lights on’
