# Supplementary material for: Peripheral immune circadian variation, synchronisation and possible dysrhythmia in established type 1 diabetes
Source: Diabetologia. 2021 May 18;64(8):1822–33. doi: 10.1007/s00125-021-05468-6 (PMC8245361; doi:10.1007/s00125-021-05468-6)
Supplement: Supplementary file 1 — (PDF 641 kb) [file 125_2021_5468_MOESM1_ESM.pdf]

ELECTRONIC SUPPLEMENTARY MATERIAL (ESM)

ESM METHODS

Murine Validation Experiment:

Animals:

Male C57BL6 male mice were obtained from the Jackson Laboratory (Bar Harbor, ME) 8 weeks of age. Mice were housed in the institutional animal care facilities at the Indiana University Medical School with strict 12h:12h, light:dark cycle. Diabetes was induced with an injection of streptozotocin (50mg/kg). Mice weighted between 20-26gr at time of injection. Animals were confirmed to be diabetic after one month of housing and when the serum glucose level was above 250 mg/dL for at least two consecutive measurements. At 4 months of age, diabetic and control mice were randomly assigned to a time point of a 24hr cycle and terminated at “Zeitgeber” times, ZT1, ZT5, ZT9, ZT13, ZT17, ZT21 (ZT= time since “lights on”).

Single cell preparation

Single cell suspensions were prepared from whole blood at the termination of the experiment. Briefly, whole blood was separated with lympholyte Mammal (Cedarlane) according to manufacturer’s instructions. Single cell suspensions were lysed with 1 ml RBC lysis reagent (ACS, Stem cells), washed twice and then stained with antibodies for flow cytometry (described in detail below).

Flow cytometry and FACS analysis

Antibodies were purchased from BD Biosciences, San Jose, CA unless otherwise stated. Combinations of the following antibodies were used for staining of blood mononuclear cells. FcγR (2.4G2); FITC-CD43 (S7); PerCPCy5.5- CD3e (145-2C11); PerCPCy5.5- B220 (RA3-6B2); PerCPCy5.5- NK1.1 (PK136); PerCPCy5.5- Ly6G (1A8); PerCPCy5.5- CD90.2 (Thy1.2); PE- CCR2 (475301, RDR systems);PECy7-Ly6C (Al-21); PE-CF594- CD45 (30-F11); APC- CD11c (HL3); APC- F4/80 (BM8); APC- IA-b (AF6-120.1); APC-efluor780-CD11b (M1/7,

eBiosciences), biotin –CD115(AFS98), fixable - viability dye eFluor450 (eBiosciences), streptavidin (eBiosciences); FITC-CD62L MEL-14), PE-CD44 (IM7), PE-CF594-CCR7 (150503), APC-CD4(RM4-5), PE-Cy7-CD8 (RPA-T8), APC-Cy7-CD127 (A7R34).

Gating: Lin1 (CD3, CD19, NK1.1) = lymphocytes; Lin2 (CD11c, F480, IA-b) = antigen presenting cells. Monocytes were defined as live/ $CD45^{+}$/ Lin1^{-} / Lin2^{-} / $CD11b^{+}$ $CD115^{+}$. Inflammatory monocytes were defined as $Ly6C^{hi}$ $CD43^{lo}$ and patrolling monocytes were defined as $Ly6C^{lo}$ $CD43^{hi}$; T cells were defined as live/CD3+ ; CD4 T cells as live/CD3+ CD4+ cells; CD8 T cells as live/CD3+CD8+cells; naïve T cells as live/ CD3+ CD4+ or CD8+ T cells/ $CD44^{lo}$ $CD62L^{hi}$ $CCR7^{hi}$; effector memory T cells as live/ CD3+ CD4+ or CD8+/ $CD44^{hi}$ $CD62L^{lo}$ $CCR7^{lo}$; central memory T cells as live/ CD3+ CD4+ or CD8+/ $CD44^{hi}$ $CD62L^{h\ i}$$CCR7^{hi}$.

Samples were fixed and data acquired on a LSR II flow cytometer equipped with 407, 488, 561 and 630nm lasers at the Indiana University School of Medicine Simon Cancer Center flow

Statistical Methods

Preparatory to analysis (data not shown), examination of ICC ("intraclass correlation coefficient", described below) values showed that percentage-based immune parameters tended to have higher ICC than count data indicating that percentage data has removed some of the within-subject circadian variation. This is a reasonable conclusion since some of the variation of the raw count data reflects between-subject variations in population numbers while the percent data factors that out, reporting results essentially as a count per standardized population count (i.e. population count=100) to yield a percent. As discussed below, we have established a rule for determining whether within-subject variation is "biologically significant" based on the ICC and therefore concluded that the use of percentage data would be a more conservative approach to selection of immune parameters for further study. We therefore restricted our primary circadian analysis to percentage data for the immune populations.

We followed study design protocols and analysis methodologies that are commonly found in human circadian studies, including sample size selection. Standardized versions of the variables were analyzed in order to increase the precision of circadian estimates by removing differences in subject-level dynamic ranges. For each subject, the measurements of each variable (cell populations, cytokine level and clock gene expression) were transformed into “z-scores“ by subtracting the mean of the variable and dividing by the standard deviation of the variable. These values can then be intrepreted as the number of standard deviations from the mean and translated back to the original, untransformed values, using the means and standardard deviations of the variable.

Circadian rhythmicity patterns were estimated using "COSINOR" analysis[43] implemented in SAS (v9.4, Cary, NC) as a mixed linear model with random subject effect. Following Cornelissen[43], a linear expression of a single phase Cosinor model of a value measured at time "t" is given by:

$$Y(t) = M + \beta x + \gamma z + e(t)$$

where

$$\beta = A cos\phi; \gamma = -A sin\phi; x = \cos\left(\frac{2\pi t}{T}\right); z = \sin\left(\frac{2\pi t}{T}\right),$$

$$A\ is\ the\ amplitude\ of\ the\ curve\ and\ \phi\ the\ acrophase.$$

In fitting the linear model above, we included a random effect for subject in order to account for the repeated measurements taken from each subject. This was accomplished using SAS Procedure "Mixed" using a “Variance Components” covariance structure. In addition, we tested whether cortisol or sex influenced circadian rhythmicity by fitting the linear model both with and without these covariates.

Results from the analysis of circadian clock genes are presented in ESM Table 3, but not provided in the main text.

Satistical significance of the circadian pattern was established using the Likelihood Ratio Test (LRT) while limiting the False Discovery Rate (FDR) to 10%. The likelihood of the data under the

alternative model (COSINOR + subject random effect) was compared to the null model (subject random effect only) with a 2 df Chi-Square statistic formed by the difference in -2Ln(likelihood) from each model. Control of the overall "False Discovery Rate (FDR)" (the proportion of false postives among the total number of positive findings) was achieved using the "FDRtool" package in R (R Core Team (2017). R: A language and environment for statistical computing. R Foundation for Statistical Computing, Vienna, Austria. URL https://www.R-project.org/). The FDR method[44] is an alternative to the Bonferroni approach for controlling the rate of False Positive findings and is more powerful in settings in which many hypotheses are tested simultaneously. In this study, we set the FDR to not exceed 10% by using q-values with threshold of 10% to demarcate “FDR-controlled, statistically significant circadian rhythmicity”. Results are presented in ESM Table 4.

The analysis of synchronization was based on Pearson correlation between pairs of immune factors. We consider Pearson correlation to be appropriate when the data have been standardized as we have done. Again, FDR was limited to 10% using the R package FDRtool with the correlations. However, in this case we used the FDR p-values computed from the package in order to gain sensitivity when determining significant correlations.

Confidence intervals on amplitude, acrophase and the differences between healthy control and subjects with type 1 diabetes were computed via bootstrapping[45], implemented in the R package boot.

Student's Paired t-test was used to determine if mean cell population frequencies at 1 am and 9 pm were biologically different at $p<0.05$ significance level.

MetaCycle version 1.2.0 , a statistical package for R enabling detection of features displaying rhythmicity in time-series data[46] was used for the analysis of mouse flow cytometry data. R studio desktop v. 1.32.5042 was used for the analysis with the meta2d function. Flow cytometry absolute values for each cell population and each group (control or STZ diabetes) were first normalized as z-scores. A p value <0.05 was considered to have significant circadian rhythmicity, shown as asterisks (*) in the heatmap.

ESM TABLES

ESM Table 1 Clock Gene Expression Arrays

Assay ID	Gene	Gene Name
Hs99999901_s1	*18s rRNA*	-
Hs01060665_g1	*ACTB*	actin beta
Hs02786624_g1	*GAPDH*	glyceraldehyde-3-phosphate dehydrogenase
Hs02800695_m1	*HPRT1*	hypoxanthine phosphoribosyltransferase 1
Hs00427620_m1	*TBP*	TATA-box binding protein
Hs00172734_m1	*CRY1*	cryptochrome circadian clock 1
Hs00901393_m1	*CRY2*	cryptochrome circadian clock 2
Hs00242988_m1	*PER1*	period circadian clock 1
Hs01007553_m1	*PER2*	period circadian clock 2
Hs00213466_m1	*PER3*	period circadian clock 3
Hs01086966_m1	*TIMELESS*	timeless circadian clock
Hs00231857_m1	*CLOCK*	clock circadian regulator
Hs00231212_m1	*NPAS2*	neuronal PAS domain protein 2
Hs00154147_m1	*ARNTL*	aryl hydrocarbon receptor nuclear translocator like
Hs00253876_m1	*NR1D1*	nuclear receptor subfamily 1 group D member 1
Hs00536545_m1	*RORA*	RAR related orphan receptor A

ESM Table 2 Baseline Comparison: HC and T1D Subjects

	Healthy Controls (n=9)				T1D (n=10)		
	Age (y)	Female (%)	BMI (kg/m^2)		Age (y)	Female (%)	BMI (kg/m^2)
**mean**	32	40	28.73		27.79	60	28.11
**min**	22		23.69		19.01		24.05
**Q1**	29.25		25.54		20.97		25.10
**median**	31.5		29.57		28.03		27.86
**Q3**	35.75		30.70		29.36		30.14
**max**	38		34.58		39.64		33.62

ESM Table 3 Clock Gene Circadian Peak Level and Time of Peak in Type 1 Diabetes Subjects.

Clock Gene	Circadian Rhythmicity	Peak (SD units)	95% CI	Time of Peak#	95% CI
ARNTL		0.18	0.01, 0.37	8:15	1:56, 19:51
CLOCK	†	0.46	0.13, 0.74	10:54	7:78, 13:48
CRY1	†	0.37	0.06, 0.64	8:04	3:94, 11:70
CRY2	†	0.45	0.13, 0.73	11:21	8:01, 14:13
NPAS2	†	0.60	0.25, 0.86	11:45	9:51, 13:61
NR1D1	†	0.61	0.29, 0.88	12:05	10:23, 14:14
PER1		0.30	0.03, 0.56	15:45	8:52, 20:50
PER2		0.26	0.03, 0.53	13:01	5:42, 18:36
PER3	†	0.73	0.42, 0.99	14:54	13:45, 16:36
RORA	†	0.58	0.26, 0.82	10:55	8:70, 13:04
TIMELESS		0.15	0.01, 0.31	17:53	3:32, 23:35

†Statistically Significant, $p<0.05$. #24 hour clock time.

ESM Table 4 Likelihood Ratio Testing of Circadian Rhythmicity. “P-value” and “q-value” results from testing for the presence of circadian rhythmicity using the Likelihood Ratio Test (LRT).

Immune Factor	LRT p-value	q-value
**granulocytes**	9.14E-05	0.00085234
**CD4- CD8-**	0.637628152	0.856139872
**monocyte**	0.006737947	0.059166664
**CD4 TEMRA**	0.778800783	0.879063579
**CD8 TEMRA**	0.182683524	0.630320561
**CD8 TN**	2.07E-10	1.93E-09
**CD4 TN**	5.84E-06	5.45E-05
**DC**	0.000500451	0.004649161
**unidentified**	1.76E-05	0.00016424
**NK**	0.116484158	0.520885907
**NK CD56dim**	0.082084999	0.433789205
**CD4 TCM**	0.386741023	0.783060639
**CD8 TCM**	0.165298888	0.606731142
**B cell**	2.63E-06	2.45E-05
**lymph mono**	5.27E-05	0.000491625
**CD4**	4.59E-09	4.28E-08
**CD4+CD8+**	0.007083409	0.062012088

**classical monocyte**	4.79E-06	4.47E-05
**TREG**	8.32E-07	7.77E-06
**NK CD56bright**	0.086293586	0.446108225
**CD8**	2.92E-08	2.73E-07
**nonclassical monocyte**	4.79E-06	4.47E-05
**NK T cell**	0.020241911	0.158903706
**CD4 TEM**	0.011678567	0.098286715
**CD8 TEM**	5.63E-11	7.88E-10
**cortisol**	1.71E-13	4.77E-12
**IFNg**	0.496585304	0.822531298
**IL-12**	0.149568619	0.582633173
**IL-2**	0.449328964	0.807460416
**IL-4**	0.70468809	0.868023197
**IL-6**	0.005799405	0.051348407

ESM FIGURES

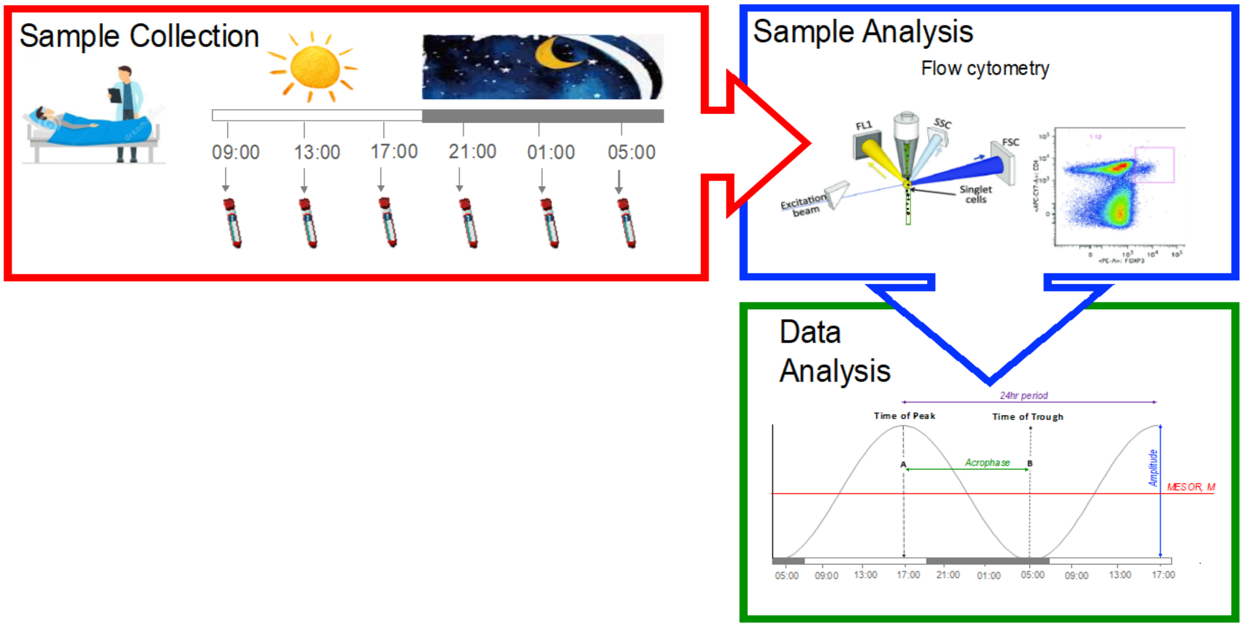

ESM Figure 1. **Schematic of the study design**. Adult volunteers with type 1 diabetes diagnosed for at least 12 months admitted for 24 hours at the Research Center University Hospital at IU. Blood samples were collected every 4 hr. at indicated times. Samples were aliquoted and one set processed immediately on site by the Flow Cytometry Resource Facility at IU. The other aliquots were shipped to the University of Florida for processing. Identified cell populations were tested for circadian rhythmicity and peak levels and times of peak and of trough were estimated for each cell population.

		mono.	temra CD4	TEM CD4	gran.	TEM CD8	nk	unident.	TEMRA CD8	cd56dim NK	nkt	CD4-CD8-	nonclass.	TCM CD4	TCM CD8	IFNγ	IL-4	IL-6	T reg.	CD4+CD8+	CD4	lymphmono.	dc	naïve CD8	CD8	B-cell	naïve CD4	cd56bright NK	class. mono.
**peak**	***ORDER***	***1***	***2***	***3***	***4***	***5***	***6***	***7***	***8***	***9***	***10***	***11***	***12***	***13***	***14***	***15***	***16***	***17***	***18***	***19***	***20***	***21***	***22***	***23***	***24***	***25***	***26***	***27***	***28***
day	**mono.**	1.00	-0.03	0.10	0.64	0.16	0.17	-0.59	-0.11	-0.11	0.31	0.30	-0.28	0.07	0.05	-0.15	-0.12	-0.18	-0.03	0.00	0.06	-0.64	0.45	-0.19	0.12	0.13	-0.04	0.11	0.28
day	**temra CD4**	-0.03	1.00	0.47	-0.30	0.31	0.38	0.16	0.44	0.35	-0.02	0.04	0.15	-0.48	-0.24	-0.07	-0.08	-0.07	-0.18	-0.16	-0.25	0.29	0.09	-0.34	-0.06	-0.33	-0.04	-0.34	-0.15
day	**TEM CD4**	0.10	0.47	1.00	-0.18	0.49	0.32	0.18	0.33	0.37	0.10	0.04	0.21	-0.45	-0.01	-0.09	-0.16	-0.10	-0.50	-0.17	-0.44	0.18	0.09	-0.55	-0.30	-0.36	-0.40	-0.36	-0.21
day	**gran.**	0.64	-0.30	-0.18	1.00	-0.18	-0.09	-0.45	-0.31	-0.23	0.02	0.09	-0.34	0.31	0.14	-0.01	0.20	0.11	0.17	0.25	0.32	-1.00	0.35	0.14	0.11	0.30	0.06	0.23	0.34
day	**TEM CD8**	0.16	0.31	0.49	-0.18	1.00	0.55	0.03	0.38	0.48	0.60	0.46	0.43	0.03	-0.01	-0.08	-0.13	0.11	-0.32	-0.17	-0.41	0.18	0.08	-0.91	-0.20	-0.31	-0.65	-0.49	-0.43
day	**nk**	0.17	0.38	0.32	-0.09	0.55	1.00	-0.01	0.76	0.83	0.53	0.33	0.42	-0.14	-0.13	-0.03	-0.04	-0.09	-0.25	-0.29	-0.47	0.08	-0.05	-0.65	-0.20	-0.48	-0.27	-0.83	-0.42
day	**unident.**	-0.59	0.16	0.18	-0.45	0.03	-0.01	1.00	0.15	0.27	-0.40	-0.50	0.32	-0.08	-0.05	0.22	0.08	0.15	-0.53	-0.13	-0.62	0.43	-0.42	-0.06	-0.68	-0.56	-0.15	-0.27	-0.32
day	**TEMRA CD**	-0.11	0.44	0.33	-0.31	0.38	0.76	0.15	1.00	0.73	0.30	0.03	0.37	-0.28	-0.26	0.20	0.02	-0.04	-0.25	-0.22	-0.49	0.30	-0.11	-0.49	-0.29	-0.60	-0.16	-0.73	-0.37
day	**cd56dim NK**	-0.11	0.35	0.37	-0.23	0.48	0.83	0.27	0.73	1.00	0.40	0.22	0.54	-0.22	-0.22	-0.01	-0.12	-0.07	-0.30	-0.32	-0.57	0.21	-0.17	-0.58	-0.38	-0.54	-0.34	-1.00	-0.54
day	**nkt**	0.31	-0.02	0.10	0.02	0.60	0.53	-0.40	0.30	0.40	1.00	0.69	0.28	0.13	0.00	-0.17	-0.30	-0.10	0.19	0.07	0.04	-0.03	0.28	-0.57	0.24	0.02	-0.34	-0.41	-0.29
day	**CD4-CD8-**	0.30	0.04	0.04	0.09	0.46	0.33	-0.50	0.03	0.22	0.69	1.00	0.39	0.00	-0.06	-0.41	-0.29	-0.03	0.23	-0.05	0.20	-0.08	0.25	-0.36	0.43	0.23	-0.25	-0.22	-0.39
day	**nonclass.**	-0.28	0.15	0.21	-0.34	0.43	0.42	0.32	0.37	0.54	0.28	0.39	1.00	-0.07	-0.01	-0.09	-0.17	0.26	-0.16	-0.18	-0.35	0.34	-0.28	-0.46	-0.24	-0.38	-0.46	-0.54	-1.00
day	**TCM CD4**	0.07	-0.48	-0.45	0.31	0.03	-0.14	-0.08	-0.28	-0.22	0.13	0.00	-0.07	1.00	0.54	0.24	0.39	0.21	0.27	0.38	0.23	-0.31	0.02	-0.02	0.07	0.25	-0.31	0.22	0.07
day	**TCM CD8**	0.05	-0.24	-0.01	0.14	-0.01	-0.13	-0.05	-0.26	-0.22	0.00	-0.06	-0.01	0.54	1.00	0.10	0.21	0.11	0.11	0.31	0.18	-0.13	0.23	-0.20	-0.07	0.28	-0.35	0.22	0.01
day	**IFNγ**	-0.15	-0.07	-0.09	-0.01	-0.08	-0.03	0.22	0.20	-0.01	-0.17	-0.41	-0.09	0.24	0.10	1.00	0.62	0.51	0.01	0.07	-0.11	0.00	-0.05	0.03	-0.23	-0.25	-0.02	0.01	0.09
day	**IL-4**	-0.12	-0.08	-0.16	0.20	-0.13	-0.04	0.08	0.02	-0.12	-0.30	-0.29	-0.17	0.39	0.21	0.62	1.00	0.57	0.17	0.08	0.10	-0.20	-0.09	0.09	-0.11	0.05	0.05	0.12	0.17
night	**IL-6**	-0.18	-0.07	-0.10	0.11	0.11	-0.09	0.15	-0.04	-0.07	-0.10	-0.03	0.26	0.21	0.11	0.51	0.57	1.00	0.10	0.08	0.00	-0.11	-0.09	-0.06	-0.08	-0.04	-0.11	0.07	-0.26
night	**T reg.**	-0.03	-0.18	-0.50	0.17	-0.32	-0.25	-0.53	-0.25	-0.30	0.19	0.23	-0.16	0.27	0.11	0.01	0.17	0.10	1.00	0.26	0.78	-0.16	0.07	0.38	0.69	0.54	0.35	0.30	0.16
night	**CD4+CD8+**	0.00	-0.16	-0.17	0.25	-0.17	-0.29	-0.13	-0.22	-0.32	0.07	-0.05	-0.18	0.38	0.31	0.07	0.08	0.08	0.26	1.00	0.43	-0.24	0.38	0.13	0.27	0.20	0.00	0.32	0.18
night	**CD4**	0.06	-0.25	-0.44	0.32	-0.41	-0.47	-0.62	-0.49	-0.57	0.04	0.20	-0.35	0.23	0.18	-0.11	0.10	0.00	0.78	0.43	1.00	-0.30	0.30	0.46	0.73	0.66	0.33	0.57	0.34
night	**lymphmono.**	-0.64	0.29	0.18	-1.00	0.18	0.08	0.43	0.30	0.21	-0.03	-0.08	0.34	-0.31	-0.13	0.00	-0.20	-0.11	-0.16	-0.24	-0.30	1.00	-0.35	-0.13	-0.10	-0.28	-0.06	-0.21	-0.34
night	**dc**	0.45	0.09	0.09	0.35	0.08	-0.05	-0.42	-0.11	-0.17	0.28	0.25	-0.28	0.02	0.23	-0.05	-0.09	-0.09	0.07	0.38	0.30	-0.35	1.00	-0.12	0.22	0.36	-0.12	0.17	0.28
night	**naïve CD8**	-0.19	-0.34	-0.55	0.14	-0.91	-0.65	-0.06	-0.49	-0.58	-0.57	-0.36	-0.46	-0.02	-0.20	0.03	0.09	-0.06	0.38	0.13	0.46	-0.13	-0.12	1.00	0.30	0.37	0.69	0.59	0.46
night	**CD8**	0.12	-0.06	-0.30	0.11	-0.20	-0.20	-0.68	-0.29	-0.38	0.24	0.43	-0.24	0.07	-0.07	-0.23	-0.11	-0.08	0.69	0.27	0.73	-0.10	0.22	0.30	1.00	0.52	0.32	0.38	0.23
night	**B-cell**	0.13	-0.33	-0.36	0.30	-0.31	-0.48	-0.56	-0.60	-0.54	0.02	0.23	-0.38	0.25	0.28	-0.25	0.05	-0.04	0.54	0.20	0.66	-0.28	0.36	0.37	0.52	1.00	0.14	0.54	0.38
night	**naïve CD4**	-0.04	-0.04	-0.40	0.06	-0.65	-0.27	-0.15	-0.16	-0.34	-0.34	-0.25	-0.46	-0.31	-0.35	-0.02	0.05	-0.11	0.35	0.00	0.33	-0.06	-0.12	0.69	0.32	0.14	1.00	0.34	0.46
night	**cd56bright**	0.11	-0.34	-0.36	0.23	-0.49	-0.83	-0.27	-0.73	-1.00	-0.41	-0.22	-0.54	0.22	0.22	0.01	0.12	0.07	0.30	0.32	0.57	-0.21	0.17	0.59	0.38	0.54	0.34	1.00	0.54
night	**class. mono.**	0.28	-0.15	-0.21	0.34	-0.43	-0.42	-0.32	-0.37	-0.54	-0.29	-0.39	-1.00	0.07	0.01	0.09	0.17	-0.26	0.16	0.18	0.34	-0.34	0.28	0.46	0.23	0.38	0.46	0.54	1.00

ESM Figure 2. Synchronization Correlations of immune-related circulatory cell populations and cytokines after ordering on the sequence of their peak appearance during a 24-hour observation period. Correlations between pairs of immune variables whose peaks occur one after the other (indicating phase synchronization) are highlighted in bold text and border and appear above/below the diagonal. Significant (FDR-adjusted) correlations are indicated by shading. Red=negative, blue=positive correlation. Time of day of peak occurrence during the day of the study is indicated in the left-most column.
